# JAMM: A Metalloprotease-Like Zinc Site in the Proteasome and Signalosome

**DOI:** 10.1371/journal.pbio.0020002

**Published:** 2003-11-24

**Authors:** Xavier I Ambroggio, Douglas C Rees, Raymond J Deshaies

**Affiliations:** **1**Division of Biology, California Institute of TechnologyPasadena, CaliforniaUnited States of America; **2**Division of Chemistry and Chemical Engineering, California Institute of TechnologyPasadena, CaliforniaUnited States of America; **3**Howard Hughes Medical Institute, Chevy ChaseMarylandUnited States of America

## Abstract

The JAMM (JAB1/MPN/Mov34 metalloenzyme) motif in Rpn11 and Csn5 underlies isopeptidase activities intrinsic to the proteasome and signalosome, respectively. We show here that the archaebacterial protein AfJAMM possesses the key features of a zinc metalloprotease, yet with a distinct fold. The histidine and aspartic acid of the conserved EX_n_HS/THX_7_SXXD motif coordinate a zinc, whereas the glutamic acid hydrogen-bonds an aqua ligand. By analogy to the active site of thermolysin, we predict that the glutamic acid serves as an acid-base catalyst and the second serine stabilizes a tetrahedral intermediate. Mutagenesis of Csn5 confirms these residues are required for Nedd8 isopeptidase activity. The active site-like architecture specified by the JAMM motif motivates structure-based approaches to the study of JAMM domain proteins and the development of therapeutic proteasome and signalosome inhibitors.

## Abstract 2

Protein structure studies suggest that deubiquitination in the proteasome is carried out by a protein with features of a zinc metalloprotease

## Introduction

Many cellular proteins are degraded by the proteasome after they become covalently modified with a multiubiquitin chain. The 26S proteasome is a massive protein composed of a 20S core and two 19S regulatory particles (Voges et al. 1999). The 20S core can be subdivided into a dimer of heptameric rings of β subunits—which contain the proteolytic active sites responsible for the protein degradation activity of the proteasome—flanked by heptameric rings of α subunits. The 19S regulatory particle can be divided into a base thought to comprise a hexameric ring of AAA ATPases and a lid composed of eight or more distinct subunits. Whereas 20S core particles and AAA ATPase rings have been found in compartmentalized proteases in prokaryotes, the lid domain of the 19S regulatory particle is unique to eukaryotes and provides the specificity of 26S proteasomes for ubiquitinated substrates (Glickman et al. 1998). Ubiquitin (Ub), an 8 kD protein, is conjugated by Ub ligases to proteasome substrates via an isopeptide bond that links its carboxyl terminus to the amino sidechain of a lysine residue in the substrate. Ub-like proteins (Ubls), of which there are several, are conjugated to their target proteins in a similar manner. Ubls typically do not promote degradation of their targets by the proteasome, but rather regulate target activity in a more subtle manner reminiscent of protein phosphorylation (Hershko and Ciechanover 1998; Peters et al. 1998).

As is the case for protein phosphorylation, the attachment of Ub and Ubls to target proteins is opposed by isopeptidase enzymes that undo the handiwork of Ub ligases. For example, removal of the Ubl Nedd8 (neural precursor cell expressed, developmentally downregulated 8) regulatory modification from the Cullin 1 (Cul1) subunit of the SCF (Skp1/Cdc53/Cullin/F-box receptor) Ub ligase is catalyzed by the COP9 signalosome (CSN) (Lyapina et al. 2001). The CSN was identified in Arabidopsis thaliana from genetic studies of constitutively photomorphogenic mutant plants (Osterlund et al. 1999). It later became evident that CSN and the proteasome lid are paralogous complexes (Glickman et al. 1998; Seeger et al. 1998; Wei et al. 1998). Csn5 of CSN and Rpn11 (regulatory particle number 11) of the proteasome lid are the subunits that are most closely related between the two complexes. CSN-dependent isopeptidase activity is sensitive to metal ion chelators, and Csn5 contains a conserved, putative metal-binding motif (EX_n_HS/THX_7_SXXD), referred to as the JAMM motif, that is embedded within the larger JAB1/MPN/Mov34 domain (hereafter referred to as the JAMM domain) and is critical for Csn5 function in vivo (Cope et al. 2002). Removal of Ub from proteasome substrates is also promoted by a metal ion-dependent isopeptidase activity associated with the proteasome (Verma et al. 2002; Yao and Cohen 2002). The JAMM/MPN^+^ motif of Rpn11 is critical for its function in vivo (Maytal-Kivity et al. 2002; Verma et al. 2002; Yao and Cohen 2002), and proteasomes that contain Rpn11 bearing a mutated JAMM motif are unable to promote deubiquitination and degradation of the proteasome substrate Sic1 (Verma et al. 2002). Taken together, these observations suggested that the JAMM motif specifies a catalytic center that in turn defines a novel family of metalloisopeptidases. Interestingly, the JAMM motif is found in proteins from all three domains of life (Cope et al. 2002; Maytal-Kivity et al. 2002), indicating that it has functions beyond the Ub system. In this study, we present the crystal structure of the *Archaeoglobus fulgidus AF2198* gene product AfJAMM and explore the implications of its novel metalloprotease architecture.

## Results and Discussion

**Figure 1:**
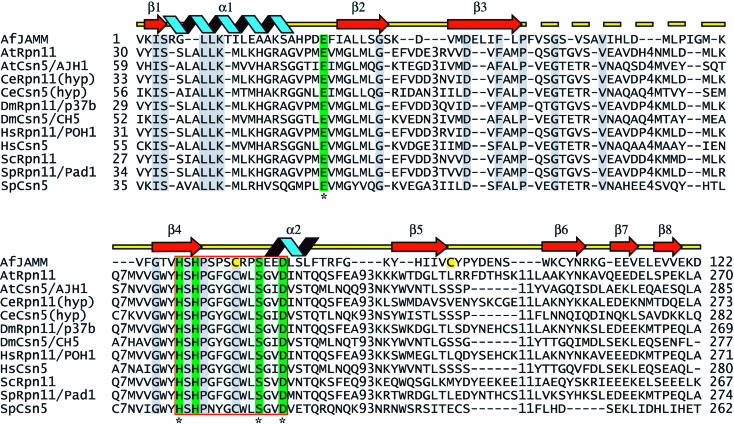
Alignment of Eukaryotic JAMM Domains with AfJAMM Eukaryotic JAMM domain proteins were aligned with AfJAMM using ClustalX and manually refined. Sequences are named with a two-letter code corresponding to the genus and species of the respective organism followed by the name of the protein (see Supporting Information for accession numbers), and ‘hyp’ is an abbreviation for hypothetical. The JAMM motif comprises the residues highlighted in green (E22, H67, H69, S77, and D80), and the active site core is surrounded by a red box. Conserved residues are highlighted in gray. The disulfide cysteine residues are highlighted in yellow (C74, C95). Active site residues that were mutated in S. pombe Csn5 are marked with an asterisk beneath the alignment. The secondary structure of AfJAMM is indicated above the sequence; helices are blue, sheets are red arrows, and loops are yellow lines. The dashed yellow line indicates a loop (F42–G58) that is disordered in the crystal.

We proposed that the subset of JAMM domain proteins that contain a JAMM motif comprise a novel family of metallopeptidases (Cope et al. 2002). To gain a clearer understanding of these putative enzymes—in particular the pertinent subunits of the proteasome lid and signalosome (Figure 1)—we cloned and expressed in Escherichia coli a variety of JAMM motif-containing proteins to find a suitable candidate for crystallographic analysis. The expression of all candidates except for AfJAMM led to insoluble aggregates. Unlike many JAMM proteins that contain an additional domain, the AfJAMM protein consists entirely of the JAMM domain. We were able to purify and crystallize native and selenomethionine-substituted AfJAMM; the latter was used for phasing by employing the multiwavelength anomalous diffraction (MAD) technique (see Table 1 for statistics).

**Table 1:**
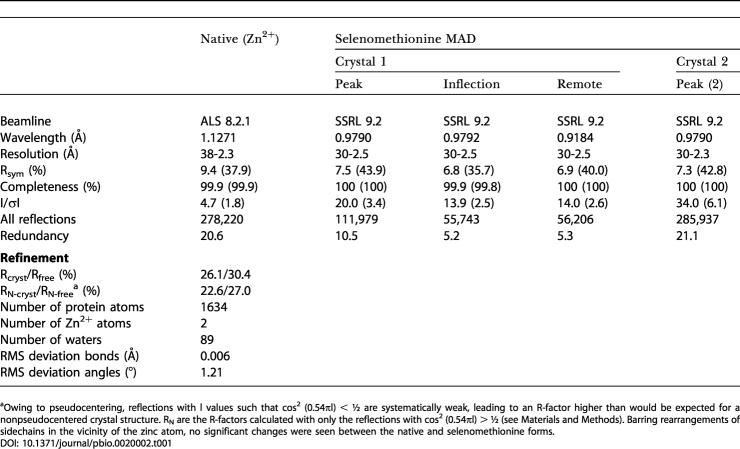
Data Collection Statistics

^a^Owing to pseudocentering, reflections with l values such that cos^2^ (0.54πl) < ½ are systematically weak, leading to an R-factor higher than would be expected for a nonpseudocentered crystal structure. R_N_ are the R-factors calculated with only the reflections with cos^2^ (0.54πl) > ½ (see Materials and Methods). Barring rearrangements of sidechains in the vicinity of the zinc atom, no significant changes were seen between the native and selenomethionine forms

**Figure 2:**
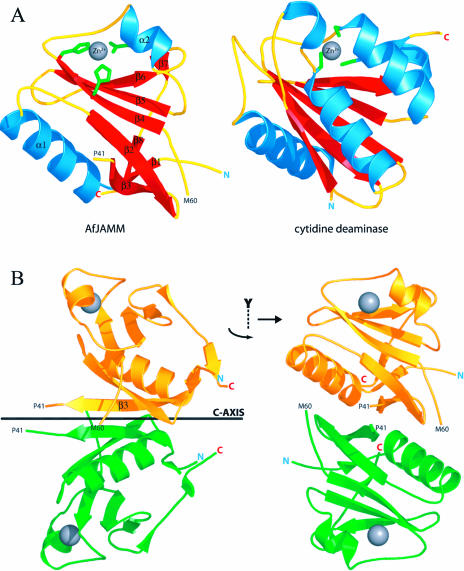
Crystal Structure of AfJAMM (A) On the left, the AfJAMM protomer is presented. The amino and carboxyl termini are marked by N and C. The catalytic zinc atom is depicted as a gray sphere. The zinc ligands (H67, H69, and D80) are colored in green. Secondary structure elements are numbered α1–α2 and β1–β8. The amino acids that mark the beginning and end of the disordered loop (P41–M60) are labeled. On the right, the crystal structure of the cytidine deaminase protomer is shown in the same orientation as AfJAMM to highlight the fold likeness as well as the similarly situated zinc-binding sites. The zinc ligands (C53, C86, and C89) are colored in green. (B) The dimer in the asymmetric unit of AfJAMM crystals. The side view is obtained by rotating the monomer in (A) by 90° as indicated by the quarter-arrow around the y-axis. The gold protomer is related to the green protomer by a 180° rotation around the crystallographic c-axis (shown as a black bar in the side view) and a translation of 3.38 Å.

AfJAMM consists of an eight-stranded β sheet (β1–β8), flanked by a long α helix (α1) between the first and second strand, and a short α helix (α2) between the fourth and fifth strand. This β sheet resembles a β barrel halved longitudinally and curled around α1 (Figure 2A). The α2 helix is oriented lengthwise on the convex surface of the β sheet. The zinc-binding site is adjacent to a loop that spans the end of β4 to the beginning of α2 and is stabilized by a disulfide bond between C74 from this loop to C95 on β5. Although disulfide bonds are scarce in intracellular proteins, they are often present in homologous proteins found in hyperthermophiles (Mallick et al. 2002). The overall fold resembles that of the zinc metalloenzyme cytidine deaminase (CDA). CDA from Bacillus subtilis (Johansson et al. 2002) can be superimposed onto AfJAMM with a root-mean squared (RMS) deviation of 3.0 Å over 79 α carbons, despite only 9% sequence identity over structurally aligned residues. The catalytic zinc ions of AfJAMM and CDA, 4.9 Å apart in the superposition, occupy the same general vicinity in the tertiary structures but are coordinated by entirely different protein ligands, two histidines and an aspartic acid in AfJAMM compared to three cysteines in CDA, located at different positions in the sequence (Figure 2A). Consequently, the JAMM fold represents a departure from the papain-like cysteine protease architecture that underlies the deubiquitinating activity of the most thoroughly characterized deubiquitinating enzymes (DUBs), the Ub carboxy-terminal hydrolases (UCHs) (Johnston et al. 1997) and Ub-specific proteases (UBPs) (Hu et al. 2002).

The two AfJAMM subunits in the asymmetric unit are connected through a parallel β sheet formed at the dimer interface (Figure 2B). The subunits are related by a 2-fold screw axis along the crystallographic c-axis with a translation of 3.38 Å, corresponding to a displacement of one residue along the β3 strand. AfJAMM behaves as a monomer during size exclusion chromatography, suggesting that the dimer observed in the asymmetric unit is an artifact of crystallization. Yet the residues of β3 are highly conserved among JAMM proteins (see Figure 1) and predominantly hydrophobic, which makes it difficult to regard the observed interaction as completely insignificant. Flanking β3 to the carboxy-terminal side, there is a striking covariation of residues, MPQSGTG in Rpn11 orthologues and LPVEGTE in Csn5 orthologues. The potential of β3 and the flanking region to mediate specific protein–protein interactions, such as the assembly of Rpn11 and Csn5 into their respective complexes or their specificity towards Ub or Nedd8, warrants further investigation.

**Figure 3:**
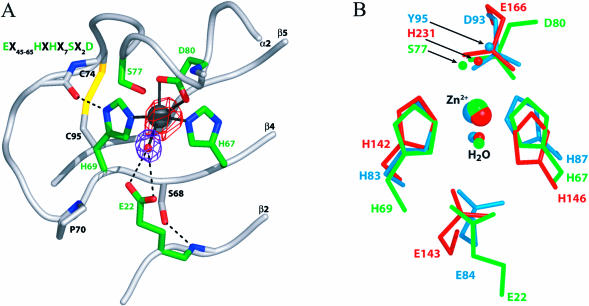
Metalloprotease-Like Active Site of AfJAMM (A) The active site of AfJAMM is shown centered around the catalytic zinc ion, which is represented as a dark gray sphere surrounded by anomalous cross Fourier difference density (contoured at 9.5 σ) colored in red. The aqua ligand, which lies at 2.9 Å from the zinc, is shown as a red sphere surrounded by purple density (contoured at 3 σ) of an F_obs_ – F_calc_ map, in which the aqua ligand was omitted from the calculation. Residues that underlie isopeptide bond cleavage are shown in green. The carboxylate oxygen atoms of D80 lie 2.2 Å from the zinc. The N^ɛ2^ atoms of H67 and H69 lie 2.1 Å from the zinc. The carboxylate oxygen atoms of E22 lie 3.2–3.5 Å from the aqua ligand and 4.5–5.0 Å from the zinc. Ancillary active site residues and the backbone (ribbon diagram) are shown in grey. The disulfide bond that links C74 to C95 is shown in yellow. The JAMM motif is shown in the upper lefthand corner for reference. (B) Superimposition of active site residues in ScNP, thermolysin, and AfJAMM. AfJAMM is in green, ScNP in blue, and thermolysin in red. For clarity only, the sidechains from the residues that bind the zinc or aqua ligands are shown in their entirety. In addition, atoms that stabilize the putative tetrahedral intermediate are shown. These include O^γ^ of S77 in AfJAMM, O^η^ of Y95 in ScNP, and the N^ɛ2^ of H231 in thermolysin.

The zinc-binding site of AfJAMM is located in a furrow formed by the convex surface of the β2–β4 sheet and α2. The catalytic zinc has a tetrahedral coordination sphere (Figure 3A), with ligands provided by N^ɛ2^ of H67 and H69 on β4, the carboxylate of D80 on α2, and a water molecule. The latter hydrogen-bonds to the sidechain of E22 on β2. Thus, the crystal structure confirms previous predictions that the histidine and aspartic acid residues in the JAMM motif are ligands for a metal (Cope et al. 2002; Verma et al. 2002; Yao and Cohen 2002). It must be noted that the identity of the physiological metal in AfJAMM and eukaryotic JAMM homologues is still unknown. The majority of metalloproteases naturally employ zinc but show altered activities with other substituted metals (Auld 1995).

The arrangement of zinc ligands in AfJAMM resembles that found in thermolysin, the Streptomyces caespitosus zinc endoprotease (ScNP), and neurolysin, a mammalian metalloprotease (Kurisu et al. 2000; Brown et al. 2001; English et al. 2001). Thermolysin, neurolysin, and ScNP are homologues that have the classical HEXXH metalloprotease motif and adopt the same core fold. In contrast, the sequence, zinc-binding motif, and fold adopted by AfJAMM are entirely distinct. Nonetheless, the active site metal and ligand atoms of thermolysin and ScNP can be superimposed on those of AfJAMM with an RMS deviation of approximately 0.4–0.5 Å (Figure 3B).

While this manuscript was under revision, an independent report of a crystal structure of the *AF2198* gene product appeared (Tran et al. 2003). These authors used the fold similarity to CDA as a framework to evaluate the function of the JAMM motif. Given the biochemical data supporting the JAMM motif's role in proteolysis, the common active site architecture seen in AfJAMM and thermolysin, and the similarity of zinc ligands between thermolysin and AfJAMM, we believe that the extensive body of mechanistic studies on thermolysin and related metalloproteases provide a better framework for the analysis of JAMM function than CDA. In addition to the correspondence between zinc ligands, the glutamic acid residue (E166) downstream of the HEXXH motif of thermolysin is functionally equivalent to the aspartic acid ligand of AfJAMM (D80). E22 in AfJAMM is functionally equivalent to the glutamic acid in thermolysin's HEXXH motif, which serves as the general acid-base catalyst. The conserved serine between the histidine ligands interacts with E22 through a sidechain–main chain hydrogen bond. In more distant JAMM relatives, the serine is replaced by a threonine or asparagine (Aravind and Ponting 1998), both of which are capable of the same bracing function. Meanwhile, the γ-hydroxyl group of the highly conserved S77 in AfJAMM occupies a position similar to N^ɛ2^ of H231 in thermolysin. This atom flanks the ‘oxyanion hole’ and is implicated in stabilizing the tetrahedral intermediate formed during hydrolysis of the scissile bond (Matthews 1988; Lipscomb and Strater 1996).

**Figure 4:**
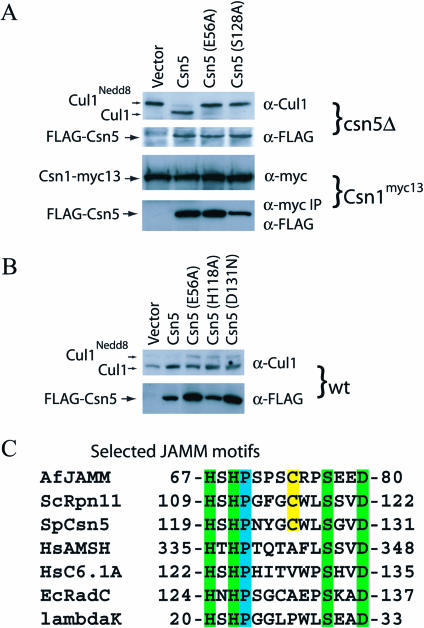
Mutations in the JAMM Motif of Csn5 Abrogate the Deneddylating Activity of the CSN (A) Mutations in the glutamic acid (E56A) that positions the aqua ligand and in the proposed catalytic serine (S128A) of Csn5 disrupt deneddylation of Cul1 by CSN but have no effect on assembly with Csn1. A *csn5Δ* strain of S. pombe was transformed with an empty pREP-41 plasmid (lane 1) or with the plasmid encoding FLAG tagged: Csn5 (lane 2), Csn5^E56A^ (lane 3), or Csn5^S128A^ (lane 4). Whole-cell lysates were used for Western blot analysis with anti-Cul1 antibodies (top gel) and anti-FLAG antibodies (second from top). A strain with a *myc13*-tagged Csn1 was transformed with the above plasmids, and whole-cell lysates were used for Western blot analysis. Antibodies to the Myc tag were used to detect Csn1*^myc13^* (third from top), and were used to pull down Csn1*^myc13^* and subsequently blot with anti-FLAG antibodies to detect coprecipitated Csn5 mutant proteins (bottom gel). (B) Mutations in the JAMM motif display a modest dominant-negative phenotype. Western blot analysis of crude cell lysates was performed as described in (A). (C) Selected JAMM motifs from proteins of diverse functions. The canonical JAMM motif residues are highlighted in green. The conserved proline is highlighted in blue, and semiconserved cysteine is highlighted in yellow.

AfJAMM was tested for the ability to hydrolyze a number of substrates, including Ub derivatives, resofurin-labeled casein, and D-alanine compounds. Unfortunately, none of the in vitro assays yielded positive results. As nothing is known about AfJAMM in the context of A. fulgidus biology, these negative results do not rule out the possibility that AfJAMM functions as a peptide hydrolase in vivo. To validate the suitability of the AfJAMM structure as a basic model for eukaryotic JAMM proteins, we performed site-directed mutagenesis of *Schizosaccharomyces pombe csn5^+^*. The zinc ligands of Csn5 were previously established to be essential for its role in sustaining cleavage of the isopeptide bond that links Nedd8 to Cul1 (Cope et al. 2002). Alanine substitutions for the putative general acid-base catalyst (E56A) and the catalytic serine (S128) in the JAMM motif of Csn5 likewise abolished its ability to remove the Nedd8 moiety from Cul1 in a *csn5* ^+^ background (Figure 4A). The E56A mutation had no effect on the assembly of Csn5 with Csn1*^myc13^*, while assembly with S128A was slightly hindered (Figure 4A). Mutation of the equivalent serine codon in *RPN11* destroyed complementing activity without altering assembly of Rpn11 into the lid. However, the effect of this mutation on Rpn11 isopeptidase activity was not evaluated (Maytal-Kivity et al. 2002). Alanine substitutions for a catalytic residue (E56) or zinc ligands (H118A, D131N) exerted a modest dominant-negative phenotype in *csn5^+^* cells (Figure 4B).

We have been able to assign biochemical functions to Csn5 and Rpn11 (Cope et al. 2002; Verma et al. 2002; Yao and Cohen 2002), but the functions of other eukaryotic JAMM proteins (Figure 4C) such as AMSH and C6.1A, as well as the prokaryotic protein RadC and the viral phage λ tail assembly protein K, remain unknown. The structure of AfJAMM provides a useful tool for dissecting the functions of JAMM motifs in these varied contexts and inspires the search for specific JAMM active site inhibitors. The mechanistic implications of the AfJAMM structure explain why the deubiquitinating activity of the lid was unaffected by inhibitors of classical DUBs, the UCHs and UBPs. In classical DUBs, the nucleophile that attacks the carbon of the scissile bond is provided by a cysteine residue in the active site. This property is exploited by using the irreversible inhibitor Ub–aldehyde, which forms a nonhydrolyzable bond to the nucleophilic cysteine (Johnston et al. 1999). In contrast, JAMM proteins likely hydrolyze Ub conjugates in a manner similar to thermolysin, in which the zinc-polarized aqua ligand serves as the nucleophile (Lipscomb and Strater 1996). In the case of thermolysin, metal chelators and phosphonamidate peptides are effective inhibitors (Bartlett and Marlowe 1987), whereas other zinc metalloproteases are sensitive to peptidomimetic substrates bearing a hydroxamate group (Skiles et al. 2001). Metal chelators have been shown to be effective inhibitors of JAMM proteins (Cope et al. 2002; Verma et al. 2002); it would be interesting to see whether phosphonamidate and hydroxamate peptide mimics of Ub conjugate isopeptides would be equally effective.

The proteasome inhibitor PS-341 has gained attention for its novelty and effectiveness in treating various forms of cancer (Adams 2002). PS-341 was recently approved by the United States' Food and Drug Administration for treatment of relapsed multiple myeloma, thereby validating the proteasome as a target for anticancer therapies. The active site of JAMM proteins is an intriguing target for second-generation therapeutics targeted at the Ub–proteasome pathway for two reasons: the JAMM motif in the proteasome lid is essential for the proteasome to function and the JAMM motif in the CSN specifically regulates the activity of a critical family of E3 Ub ligases (Nalepa and Harper 2003). Inhibition of SCF and other Cullin-based ligases by way of the JAMM motif may be a more specific means of modulating levels of key proteasome substrates in cancer cells.

## Materials and Methods

The gene for A. fulgidus JAMM (Ponting et al. 1999), open reading frame *AF2198*, was cloned from genomic DNA (ATCC #49558D; American Type Culture Collection, Manassas, Virginia, United States) into the pCRT7 vectors (Invitrogen, Carlsbad, California, United States). During cloning, the alternate start codon, GTG, was replaced with the canonical start codon, ATG. The construct was expressed in BL21(DE3)pLysS cells (Novagen, Madison, Wisconsin, United States). The cells were grown to midlog phase in terrific broth media and induced with 0.5 mM IPTG. The cells were lysed by sonication and the protein was isolated by immobilized metal ion chromatography using a Ni-NTA resin (Qiagen, Valencia, California, United States). The protein was further purified by gel filtration on a Sephacryl S100 column (Amersham Pharmacia Biotech, Chalfont St Giles, United Kingdom) and concentrated. The amino-terminal tag was removed by limited digestion with trypsin. Mass spectrometry analysis revealed that trypsin only cut AfJAMM in the amino-terminal tag region, and only a single band was evident on a Coomassie-stained polyacrylamide gel. The tag and uncut protein were removed with Ni-NTA resin followed by anion-exchange chromatography with SOURCE 30Q resin (Amersham Pharmacia Biotech). The processed protein was then concentrated to approximately 30 mg/ml by ultrafiltration. The selenomethionine protein was produced as described elsewhere (Van Duyne et al. 1993) and purified using the same protocol as for the native protein.

Protein crystals were obtained in 100 mM NH_4_H_2_PO_4_, 200 mM sodium citrate (pH 5) using vapor diffusion with sitting drops and hanging drops. Crystals were incubated for approximately 1 min in a cryo-solution of equal volumes of reservoir solution and 35% meso-erythritol for the selenomethionine crystals and supplemented with 5 mM ZnCl_2_ for the native crystals. The crystals belonged to the space group P6_5_, with cell dimensions of a = b = 76 Å, c = 94 Å and two subunits per asymmetric unit. Data for the selenomethionine crystals were collected on Beamline 9.2 at the Stanford Synchrotron Radiation Laboratory (SSRL) (Stanford, California, United States) and data for the native crystals were collected on Beamline 8.2.1 at the Advanced Light Source (ALS) (Lawrence Berkeley National Laboratory, Berkeley, California, United States) (see Table 1).

Phases were obtained by the MAD technique using data collected from selenomethionine-substituted crystals (see Table 1). Three Se atoms were located by SOLVE (Terwilliger and Berendzen 1999) and used to calculate the initial phases. Phasing was subsequently improved by noncrystallographic symmetry averaging, using operators derived from the Se positions, and solvent flattening in RESOLVE (Terwilliger 2000). The polypeptide model was built in O (Jones et al. 1991) and refined with CNS (Brünger et al. 1998).

Since two monomers in the unit cell are related by a fractional translation along c of approximately 0.54, the intensities of the diffraction pattern are modulated by a factor of cos^2^ (0.54πl). As a result, reflections with l-indices such that cos^2^ (0.54πl) < ½ are systematically weak, leading to an R-factor higher than would be expected for a nonpseudocentered crystal structure. However, when only the reflections with cos^2^ (0.54πl) > ½ (which will have a more normal intensity distribution) are used for the R-factor calculation, reasonable values for R are obtained.

The geometry of the final model was analyzed with PROCHECK (Morris et al. 1992). The Ramachandran plot shows 98.9% of the residues in the allowed regions and 1.1% in the disallowed regions. The main chain of K66, which constitutes the residue in the disallowed region, was modeled on segments taken from well-refined, high-resolution structures. The Protein Data Bank was searched for structural neighbors of AfJAMM using the DALI server (Holm and Sander 1993). The superpositions with cytidine deaminase (1JTK), thermolysin (1FJQ), and ScNP (1C7K) were done using the LSQKAB program of the CCP4 distribution (CCP4 1994). All structural figures were made with PyMOL (DeLano 2000).The experiments with S. pombe were performed as previously described by Cope et al. (2002).

## Supporting Information

### Accession Numbers

The accession numbers for the proteins discussed in this paper are 20S proteasomes (PDB ID 1RYP), AfJAMM (Entrez Protein ID NP_071023; PDB ID 1R5X), AMSH (Entrez Protein ID NP_006454), AtCSN5/AJH1 (Entrez Protein ID NP_173705), AtRpn11 (Entrez Protein ID NP_197745), C6.1A (Entrez Protein ID NP_077308), CeCSN5 (Entrez Protein ID NP_500841), CeRpn11 (Entrez Protein ID NP_494712), Csn5 (Entrez Protein ID NP_593131), Cul1 (Entrez Protein ID NP_594259), cytidine deaminase (PDB ID 1JTK), DmCsn5/CH5 (Entrez Protein ID NP_477442), DmRpn11/p37b (Entrez Protein ID AAF08394), EcRadC (Entrez Protein ID NP_418095), HsAMSH (Entrez Protein ID NP_006454), HsC6.1A (Entrez Protein ID NP_077308), HsCsn5 (Entrez Protein ID NP_006828), HsRpn11/POH1 (Entrez Protein ID NP_005796), JAB1 (Entrez Protein ID AAC17179), lambdaK (Entrez Protein ID AAA96551), Mov34 (Entrez Protein ID NP_034947), Mpr1p (Entrez Protein ID AAN77865), Nedd8 (Swiss-Prot ID Q15843), neurolysin (PDB ID 1I1I), Pad1p (Entrez Protein ID NP_594014), phage λ tail assembly protein K (Entrez Protein ID AAA96551), RadC (Entrez Protein ID NP_418095), Rpn11 (Entrez Protein ID AAN77865), SCF (PDB ID 1LDK), ScNP (PDB ID 1C7K), ScRpn11 (Entrez Protein ID AAN77865), Sic1 (Entrez Protein ID 1360441), SpCsn5 (Entrez Protein ID NP_593131), SpRpn11/Pad1 (Entrez Protein ID NP_594014), thermolysin (PDB ID 1FJQ), ubiquitin (Swiss-Prot ID P04838), UBP (PDB ID 1NB8), and UCH (PDB ID 1UCH).

These databases may be found at http://www.ncbi.nlm.nih.gov/entrez/ (Entrez Protein), http://www.rcsb.org/pdb/ (Protein Data Bank [PDB]), and http://us.expasy.org/sprot/ (Swiss-Prot).

## Abbreviations

AfJAMM: A. fulgidus JAMM protein
AMSH: associated molecule with SH3 domain of STAM
CDA: cytidine deaminase
CSN: COP9 signalosome
Cul1: Cullin 1
DUB: deubiquitinating enzyme
JAMM: JAB1/MPN/Mov34 metalloenzyme
MAD: multiwavelength anomalous diffraction
MPN: Mpr1p Pad1p N-terminal domain
Nedd8: neural precursor cell expressed
RMS: root-mean squared
Rpn11: regulatory particle number 11
SCF: Skp1/Cdc53/Cullin/F-box receptor
ScNP: S. caespitosus zinc endoprotease
Ub: ubiquitin
Ubls: ubiquitin-like proteins
UBP: ubiquitin-specific protease
UCH: ubiquitin C-terminal hydrolase
